# Option generation in decision-making research: why just talk?

**DOI:** 10.3389/fpsyg.2013.00728

**Published:** 2013-10-11

**Authors:** Oren Kolodny

**Affiliations:** Department of Zoology, Tel Aviv UniversityTel Aviv, Israel

**Keywords:** options, option generation, decision making, cognitive modeling, experimental design, reinforcement learning, computational modeling

Kalis et al. (2013) provide a lucid and useful discussion of options and option generation, and argue persuasively that these constructs can be instrumental in our understanding of decision making. I cannot agree more, but we must not be confused: Kalis et al. tell us what option generation is, but not how options are generated. To make progress, we must now build on these concepts to phrase the questions and specify the requirements that a cognitive model must fulfill; we need to embody our insights and hypotheses about the cognitive mechanisms into computational, and preferably also algorithmic, models. This will force us to bring into the open our implicit assumptions and postulates and will allow us to test hypotheses, to derive predictions and to plan experiments targeted at filling the gaps in our understanding.

As an illustration of the issue at hand, consider the definition by Kalis et al. of options as “representations of candidates for goal-directed actions.” A model based on this definition must include an internal representation of actions and of goals, of the relevant aspects of the world with which the agent may interact, and these interactions' predicted outcomes. This representation must support option generation, which requires an algorithm that can access the world representation, perhaps accepting as input the momentary state of the agent, and produce meaningful sequences of actions.

Representations and algorithms suitable for this purpose have been proposed in the past (e.g., Botvinick et al., 2009; Dayan, 2012). In particular, we (Kolodny et al., in review, in preparation) propose that such a representation should take the form of a hierarchical directed graph, whose vertices represent significant units in various modalities—for example, objects, actions, or locations—and whose edges represent probabilistic associations among them. Such a representation lends itself in a straightforward manner to option generation: the agent can generate and run “internal simulations” by probabilistically following trajectories along the graph's edges, each trajectory representing what the agent perceives as an optional sequence of events and actions in the world. Vertices in the graph are assigned value, providing necessary data for the decision-making process.

This approach allows us to address the questions raised by Kalis et al.: how might constraints in the option generation process be applied? What can account for differences among individuals? It also leads us to recognize other unknowns that may go unnoticed unless an algorithmic solution is sought. Some such questions are: What is represented by the agent? What objects in the world should be tracked and what actions do they afford? How are these organized? It may be difficult to answer these questions, but answering them is crucially important for understanding option generation and decision making, as well as other cognitive processes.

Our modeling must be based on insights, derived from experimentation, with the two constantly influencing and inspiring one another. I would like to note two methodological pitfalls that we must be wary of. The first is the potential confusion between descriptions and explanations: naming a phenomenon sometimes creates an illusion that it is better understood (McDermott, 1981). For example, individual differences in the hypothesized personality trait “openness to experience” may lead us to wonder, as Kalis et al. suggest, about differences among individuals in constraints on option generation; we must, however, bear in mind that our understanding of the cognitive processes behind this personality trait remains as scarce as our understanding of the option generation process. Linking one unknown to another is intriguing, but even if a link is found, we would still be short of an explanation.

The second pitfall is in the implicit assumptions behind, or the suggestive nature of, the questions we ask. For example, asking “What determines whether options are generated from memory, through automatic perceptual processes, or by creative cognition?” might suggest that these three options are distinct cognitive processes. Even if we do not intend to suggest so, and if the fallacy of this assumption is currently clear to most readers, our discourse may shape our thoughts and with time this clarity might be lost. Similarly, our choice of exploration space might channel us toward a specific, perhaps incorrect, conclusion. For example, Kalis et al. exclude what seem to be “automated” choices of action from the scope of exploration of decision making. Such a research agenda is not very likely to allow us to reach the conclusion that the same cognitive processes underlie all action choices, even though that might be the case^1^. An exploration of the full range of situations in which an action is carried out, using the same concepts and practices to analyze all of them, is likely to provide us with the unbiased data that we need to infer their underlying processes.

An important lesson can be learned from the type of definitions used in the study of animal behavior, where we are constantly reminded that we have no direct access to the cognitive “black box” of our subjects. This is the case with human subjects as well, despite the illusion to the contrary (reinforced by our own consciousness and the possibility of debriefing subjects, for example). It is instrumental to base our questions and definitions on directly observable criteria, and to phrase them in terms that do not include cognitive elements or processes, as these are themselves the objects of exploration (Heyes, 1994; Hoppitt and Laland, 2008).

Kalis et al. offer applicable definitions and insights into the nature of option generation and its critical role in decision making. I believe that these, coupled with a principled study paradigm and explicit construction of candidate cognitive models, may lead us to a better understanding of our selves.

## Footnotes

^1^ It is ironic that the example that Kalis et al. provide for an “automatic” action—the tendency to automatically read a written word which we are presented with—is actually, as they note, quite arguable, as multilingual readers clearly do need to apply a decision-making process in order to read a word successfully.
